# Blockade of 67-kDa Laminin Receptor Facilitates AQP4 Down-Regulation and BBB Disruption via ERK1/2-and p38 MAPK-Mediated PI3K/AKT Activations

**DOI:** 10.3390/cells9071670

**Published:** 2020-07-11

**Authors:** Ji-Eun Kim, Hana Park, Ji-Eun Lee, Tae-Cheon Kang

**Affiliations:** 1 Department of Anatomy and Neurobiology, College of Medicine, Hallym University, Chuncheon 24252, Korea; jieunkim@hallym.ac.kr (J.-E.K.); M19050@hallym.ac.kr (H.P.); 20183533@hallym.ac.kr (J.-E.L.); 2 Institute of Epilepsy Research, College of Medicine, Hallym University, Chuncheon 24252, Korea

**Keywords:** 3CAI, astrocyte, endothelial cell, piriform cortex, SB202190, SMI-71, U0126, wortmannin

## Abstract

Recently, we have reported that dysfunctions of 67-kDa laminin receptor (67LR) induced by status epilepticus (SE, a prolonged seizure activity) and 67LR neutralization are involved in vasogenic edema formation, accompanied by the reduced aquaporin 4 (AQP4, an astroglial specific water channel) expression in the rat piriform cortex (PC). In the present study, we found that the blockade of 67LR activated p38 mitogen-activated protein kinase (p38 MAPK) and extracellular signal-regulated kinase 1/2 (ERK1/2) signaling pathways, which enhanced phosphatidylinositol 3 kinase (PI3K)/AKT phosphorylations in endothelial cells and astrocytes, respectively. 67LR-p38 MAPK-PI3K-AKT activation in endothelial cells increased vascular permeability. In contrast, 67LR-ERK1/2-PI3K-AKT signaling pathways in astrocytes regulated astroglial viability and AQP4 expression. These findings indicate that PI3K/AKT may integrate p38 MAPK and ERK1/2 signaling pathways to regulate AQP4 expression when 67LR functionality is reduced. Thus, we suggest that 67LR-p38 MAPK/ERK1/2-PI3K-AKT-AQP4 signaling cascades may mediate serum extravasation and AQP4 expression in astroglio-vascular systems, which is one of the considerable therapeutic targets for vasogenic edema in various neurological diseases.

## 1. Introduction

Status epilepticus (SE, a prolonged seizure activity) is one of the neurologic emergencies that lead to death or permanent neurologic injury. As SE causes 3–5% of symptomatic epilepsy (~35% of epileptic syndromes), it is a high-risk factor for developing acquired epilepsy [1]. SE results in neuronal damage and astroglial death, which trigger long-term and profound alterations in the neuronal network that leads to the development of temporal lobe epilepsy (TLE) [2,3,4,5]. Furthermore, SE leads to the leakage of blood serum components into the parenchyma (referred to as vasogenic edema) across the blood–brain barrier (BBB) leading to the impaired astrocyte function and the altered potassium homeostasis. This vasogenic edema formation evokes neuroinflammation and paroxysmal neuronal discharge, which play an important role in epileptogenesis [6,7].

The BBB maintains the brain microenvironment to ensure proper nervous system functions by segregating the systemic environment. The BBB is formed by the endothelial cells lining the blood vessels, astrocytic endfeet surrounding the blood vessels and pericytes embedded in the basement membranes between the endothelial cells and the astrocytes. Astrocytes envelop >99% of the BBB endothelium and play an important role in inducing and maintaining BBB. Thus, BBB disruption is relevant to endothelial damage and reduction in the number of astroglial endfeet attached to microvasular surfaces [8,9]. Aquaporin 4 (AQP4, an astroglial specific water channel) maintains the osmolality in the brain parenchyma and attenuates vasogenic edema through blood-derived water elimination, although it is not directly involved in serum extravasation [10,11]. Indeed, SE-induced serum extravasation is relevant to the astroglial loss and the reduced AQP4 expression in the rat piriform cortex (PC) [12].

**Figure 1:**
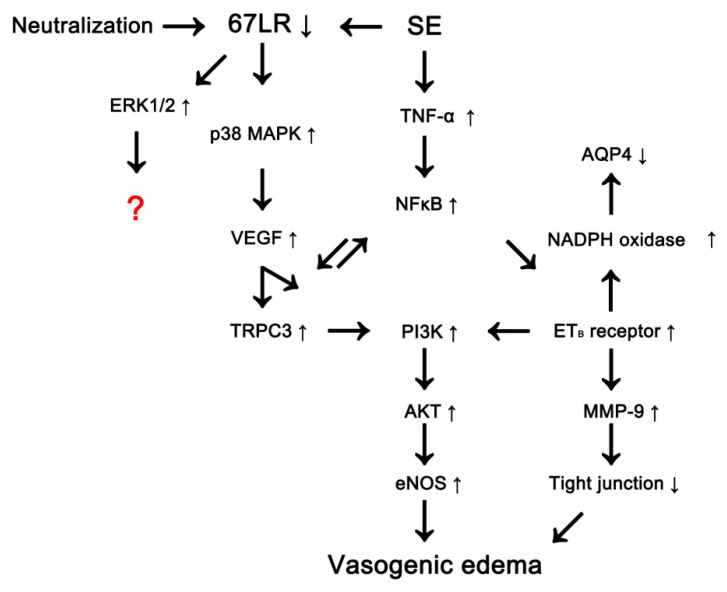
Scheme of vasogenic edema formation.

Despite the frequent occurrence of serum extravasation and its undesirable consequences, the molecular mechanisms underlying vasogenic edema formation are still unknown. In previous studies, we have reported the possible signaling pathways concerning vasogenic edema formation. Following SE, tumor necrosis factor (TNF)-α phosphorylates nuclear factor-κB (NFκB) p65-Thr435, which induces endothelin-1 (ET-1) expression in endothelial cells and, subsequently activates endothelial nitric oxide synthase (eNOS). In addition, TNF-α-induced ET-1 increases intracellular reactive oxygen species by NADPH oxidase production in astrocytes. Therefore, both TNF-α/NFκB/ET-1-mediated signalings increase vascular permeability, leading to vasogenic edema [13]. Furthermore, transient receptor potential canonical channel-3 (TRPC3) links p38 mitogen-activated protein kinase (MAPK)/vascular endothelial growth factor (VEGF) pathway to NFκB/ET_B_ receptor axis through phosphatidylinositol 3 kinase (PI3K)/AKT/eNOS signaling pathway during vasogenic edema formation (Figure 1) [14,15,16]. Recently, we have reported that SE-induced 67-kDa laminin receptor (67LR) down-regulation is involved in BBB disruption and vasogenic edema formation via activating p38 MAPK/VEGF axis concomitant with the decreased AQP4 expression in the rat PC (Figure 1) [17,18].

The 67LR is the first non-integrin laminin receptor identified by binding to immobilized laminin-1 [19]. 67LR is involved in cell proliferation, protein synthesis, cell survival, cell adhesion, and migration [20]. In addition, 67LR stabilizes or modulates the binding of laminin to other receptors [21,22]. Similar to SE, 67LR neutralization leads to serum extravasation in the PC. 67LR IgG infusion increases extracellular signal-regulated kinase 1/2 (ERK1/2) phosphorylation and induces AQP4 down-regulation under physiological condition [17,18]. Therefore, it is likely that the increased ERK1/2 activity may participate in the down-regulation of AQP4 expression, which would affect the severity of vasogenic edema formation induced by 67LR dysfunctions. However, the role of ERK1/2 in AQP4 regulation has been still unclear. Briefly, ERK1/2 activation decreases AQP4 expression following scratch-injury in cultured astrocyte [23], while it enhances AQP4 expression induced by oxygen-glucose deprivation [24]. Furthermore, Salman et al. [25] described that ERK1/2 does not affect AQP4 expression in primary human astrocytes. Thus, elucidating the 67LR-mediated regulatory mechanisms of AQP4 expression is noteworthy to understand the role of astroglio-vascular interactions in the brain, which have been elusive. Interestingly, PI3K/AKT and ERK1/2 pathways show the reciprocal regulations including cross-inhibition and cross-activation [26,27,28]. Furthermore, ERK1/2 activation enhances AKT phosphorylation in astrocytes, which down-regulates AQP4 expression [29]. Therefore, during this study, we investigated whether blockade of 67LR induced by SE or its neutralization affects PI3K/AKT and ERK1/2 signaling pathways, which would be related to vasogenic edema and AQP4 regulation.

Here, we demonstrate that the blockade of 67LR activated p38 MAPK and ERK1/2 signaling pathways, which elevated PI3K/AKT activities in endothelial cells and astrocytes, respectively. p38 MAPK-PI3K-AKT activation in endothelial cells increased vascular permeability. In contrast, ERK1/2-PI3K-AKT signaling pathways in astrocytes regulated astroglial viability and AQP4 expression. Therefore, our findings suggest that 67LR-p38 MAPK/ERK1/2-PI3K-AKT-AQP4 signaling cascades may be one of the effective therapeutic targets for the treatment of vasogenic edema.

## 2. Materials and Methods

### 2.1. Experimental Animals and Chemicals

Adult male Sprague-Dawley (SD) rats (7 weeks old) were used in the present study. Animals were housed in an acclimatized room (temperature, 22 ± 2 °C; humidity, 55 ± 5%; a 12-h light/dark cycle). The animals had access to water and food ad libitum. All efforts were made to reduce the number of animals and to minimize their suffering. Animal procedures were approved by the Institutional Animal Care and Use Committee of Hallym University (Chuncheon, South Korea, Hallym 2017-54, 19th February 2017 and Hallym 2018-2, 26th April 2018). All reagents were obtained from Sigma-Aldrich (St. Louis, MO, USA), except as noted.

### 2.2. Surgery

Under Isoflurane anesthesia (3% induction, 1.5–2% for surgery and 1.5% maintenance in a 65:35 mixture of N_2_O:O_2_), animals were infused each chemical into the right lateral ventricle (1 mm posterior; 1.5 mm lateral; −3.5 mm depth to the bregma) with a brain infusion kit 1 and an Alzet 1003D or Alzet 1007D osmotic pump (Alzet, Cupertino, CA, USA) for 3- and 7 days, respectively.

Alzet 1003 osmotic pump contained (1) control IgG (Abcam, #ab37415, UK, 50 ug/mL) + vehicle, (2) anti-67LR IgG (Abcam, #133645, UK, 50 ug/mL) + vehicle, (3) anti-67LR IgG + SB202190 (a p38 MAPK inhibitor, 0.3 mg/mL), (4) anti-67LR IgG + wortmannin (a PI3K inhibitor, 0.1 nmol), (5) anti-67LR IgG + 3-chloroacetyl indole (3CAI, an AKT inhibitor, 25 μM), and (6) 67LR IgG + U0126 (an ERK1/2 inhibitor, 25 μM). Three days after surgery (infusion), animals were used for Western blot and immunohistochemistry.

Alzet 1007D osmotic pump contained (1) vehicle, (2) SB202190 (0.3 mg/mL), (3) wortmannin (0.1 nmol), (4) 3CAI (25 μM), and (5) U0126 (25 μM). In our previous studies [14,16,17,18,30,31], each treatment did not show behavioral and neurological defects and could not change the seizure susceptibility and seizure severity in response to pilocarpine. Three days after surgery, this group was induced with SE by lithium chloride (LiCl)-pilocarpine.

### 2.3. SE Induction

To induce SE, animals were pretreated with an intraperitoneal injection of LiCl (127 mg/kg, i.p.) and atropine methylbromide (5 mg/kg, i.p.) 24 h and 20 min before pilocarpine (30 mg/kg, i.p.) treatment, respectively. Control animals received an equal volume of normal saline instead of pilocarpine. Two hours after SE, animals received diazepam (Valium; Roche, Neuilly sur-Seine, France; 10 mg/kg, i.p.) to terminate SE. Three days after SE, animals were used for immunohistochemistry and Western blot.

### 2.4. Tissue Processing

Under urethane anesthesia (1.5 g/kg, i.p.), animals were perfused via a cannula into the left ventricle of the heart with 0.9% saline followed by 4% paraformaldehyde in 0.1 M phosphate buffer (PB, pH 7.4). After perfusion, the brains were removed and post-fixed in the same fixative overnight, subsequently cryoprotection with 30% sucrose/0.1 M PBS. Brain coronal sections of 30 μm were obtained with a cryo-microtome. For western blot, the PC was dissected out, after animals were sacrificed via decapitation. The PC was rapidly removed and homogenized in lysis buffer: 50 mM Tris containing 50 mM HEPES (pH 7.4), ethylene glycol tetraacetic acid (EGTA, pH 8.0), 0.2% Tergitol type NP-40, 10 mM ethylenediaminetetraacetic acid (EDTA, pH 8.0), 15 mM sodium pyrophosphate, 100 mM β-glycerophosphate, 50 mM NaF, 150 mM NaCl, 2 mM sodium orthovanadate, 1 mM phenylmethylsulfonyl fluoride (PMSF), and 1 mM dithiothreitol (DTT) containing protease inhibitor cocktail (complete, Roche Applied Sciences, Penzberg, Bavaria, Germany) and phosphatase inhibitor cocktail (PhosSTOP^®^, Roche Applied Science, Penzberg, Bavaria, Germany). The protein concentration in the supernatant was determined using a Micro BCA Protein Assay Kit (Pierce Chemical, Dallas, TX, USA).

### 2.5. Western Blot

Western blot was performed by the standard protocol. Briefly, sample proteins (10 μg) were separated on a Bis-Tris sodium dodecyl sulfate-poly-acrylamide electrophoresis gel (SDS-PAGE). Separated proteins then were transferred to polyvinylidene fluoride membranes. The membranes were incubated with a relatively specific primary antibody (Table 1). The ECL Kit (GE Healthcare Korea, Seoul, South Korea) was used to detect signals. The bands were detected and quantified on ImageQuant LAS4000 system (GE Healthcare Korea, Seoul, South Korea). β-Actin antibody was used as a loading control for the quantitative analysis of relative expression levels of proteins. The ratio of phosphoprotein to total protein was described as the phosphorylation ratio. Thereafter, the density value of each sample obtained from 67LR IgG-infused animals (67LR IgG) and SE-induced animals (SE) was compared to that obtained from control IgG-infused animals (Cont IgG) and non-SE-induced animals (Control), respectively.

### 2.6. Immunohistochemistry

Standard procedures for immunohistochemistry were used to detect serum extravasation. Briefly, free-floating sections were washed 3 times in PBS (0.1 M, pH 7.3). Next, to inactivate the endogenous peroxidase, sections were incubated in 3% H_2_O_2_ and 10% methanol in PBS (0.1 M) for 20 min at room temperature. Later, sections were incubated in biotinylated rat IgG and ABC complex (Vector, #PK-6100, Burlingame, CA, USA, diluted 1:200). Tissue sections were developed in 3,3′-diaminobenzidine in 0.1 M Tris buffer and mounted on gelatin-coated slides. Some sections were incubated with a cocktail solution containing the primary antibodies (Table 1) in PBS containing 0.3% Triton X-100 overnight at room temperature. Thereafter, sections were visualized with appropriate Cy2- and Cy3-conjugated secondary antibodies. GFAP and SMI-71 were used for the markers of astrocytes and endothelial cells, respectively. To establish the specificity of the immunostaining, a negative control test was carried out with pre-immune serum instead of the primary antibody. No immunoreactivity was observed for the negative control in any structures. All experimental procedures in this study were performed under the same conditions and in parallel. Immunoreaction was observed using an Axio Scope microscope (Carl Zeiss Korea, Seoul, Korea).

### 2.7. Measurements of Serum Extravasation and the Volume of GFAP-Deleted Lesion

Serum extravasation was measured, as previously described [14,17,18,31]. Aforementioned, sections were incubated in biotinylated rat IgG and ABC complex (Vector, #PK-6100, Burlingame, CA, USA, diluted 1:200). Tissue sections were developed in 3,3′-diaminobenzidine in 0.1 M Tris buffer and mounted on gelatin-coated slides. Thereafter, sections (10 sections per each animal) were captured, and areas of interest were selected. Then, the measurement of serum extravasation was performed on 5× images using AxioVision Rel. 4.8 software. Serum extravasation (rat IgG immunodensity) measurements were represented as the number of a 256 grayscale. Intensity values were corrected by subtracting the average values of background noise (mean background intensity) obtained from five image inputs. Intensity of each section was standardized by setting the threshold level (mean background intensity obtained from five image inputs). Manipulation of the images was restricted to threshold and brightness adjustments to the whole image. The volume of GFAP-deleted lesion in the PC was measured by AxioVision Rel. 4.8 software and estimated by the modified Cavalieri method: *V* = Σ*area* × section thickness (30 μm) × 1/the fraction of the sections (1/6). The volumes are reported in mm^3^.

### 2.8. Statistical Analysis

The animal number (n) of each experimental group used for the evaluation was seven. The data obtained from each animal (different samples from the same experiment) were analyzed. Quantitative data were expressed as mean ± standard error of the mean. After evaluating the values on normality using Shapiro–Wilk *W*-test, data are analyzed by one-way ANOVA followed by Newman–Keuls post-hoc test. *p* < 0.05 was considered to be statistically different.

## 3. Results

### 3.1. The Effects of 67LR Neutralization on AQP4 Expression and Phosphorylations of p38 MAPK, PI3K/AKT, and ERK1/2

**Figure 2:**
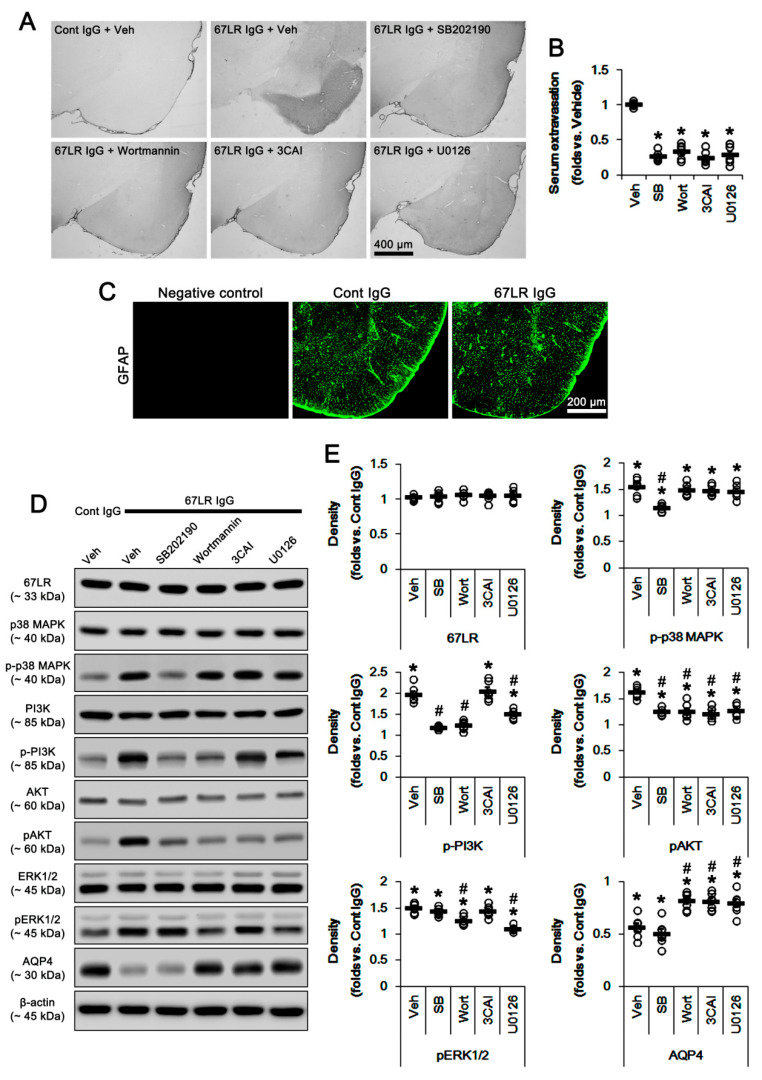
Effects of kinase inhibitors on serum extravasation, protein phosphorylations, and AQP4 expression in the PC following control IgG (Cont IgG) and 67LR neutralization (67LR IgG). 67LR IgG infusion does not evoke astroglial degeneration in the PC. Although 67LR IgG infusion does not change 67LR expression level, 67LR neutralization leads to the up-regulation of p-p38 MAPK, pPI3K-Y458, pAKT-T308, and pERK1/2 level. However, 67LR IgG infusion reduces AQP4 expression. SB202190 co-treatment attenuates serum extravasation and phosphorylations of p38 MAPK and PI3K/AKT, but not pERK1/2 and AQP4 levels. Wortmannin, 3CAI, and U0126 diminish serum extravasation. Wortmannin and U0126 inhibit the increased p-PI3K/AKT and pERK1/2 levels. 3CAI ameliorates only pAKT levels. Wortmannin, 3CAI, and U0126 increase the AQP4 expression level without affecting p38 MAPK phosphorylation. (**A**) Representative photographs for serum extravasation in the PC. **(B**) Quantitative values (mean ± S.E.M) of serum extravasation SE (*n* = 7, respectively). Open circles indicate each individual value. Horizontal bars indicate the mean value. Error bars indicate S.E.M. Significant differences are * *p* < 0.05 vs. vehicle (one-way ANOVA). (**C**) Representative photographs of GFAP expression in the PC. (**D**) Representative Western blot images for expressions and phosphorylations of 67LR, p38 MAPK, PI3K, AKT, ERK1/2, and AQP4. (**E**) Quantitative values (mean ± S.E.M) of the Western blot data concerning expressions and phosphorylations of 67LR, p38 MAPK, PI3K, AKT, ERK1/2, and AQP4 (*n* = 7, respectively). Open circles indicate each individual value. Horizontal bars indicate the mean value. Error bars indicate S.E.M. Significant differences are *^,#^ *p* < 0.05 vs. control IgG and vehicle, respectively (one-way ANOVA).

Consistent with our previous studies [5,6], 67LR IgG infusion evoked serum extravasation in the PC (*p* < 0.05 vs. control IgG, one-way ANOVA; *n* = 7, respectively; Figure 2A,B). 67LR neutralization did not result in astroglial loss in this region (Figure 2C). The blockade of 67LR expression increases p38 MAPK and PI3K/AKT activities [17,18,32]. Furthermore, p38 MAPK is one of the up-stream molecules to activate PI3K/AKT that are one of the signal transductions developing vasogenic edema formation induced by SE [14,16]. Thus, we explored if 67LR neutralization affects their activities in the PC. Although 67LR IgG infusion did not change 67LR expression level, 67LR neutralization led to up-regulation of p38 MAPK phosphorylation in the PC (*p* < 0.05 vs. control IgG, one-way ANOVA; *n* = 7, respectively; Figure 2D,E and Figure S1; Table 2). 67LR IgG significantly also increased pPI3K-Y458 and pAKT-T308 phosphorylations (*p* < 0.05 vs. control IgG, one-way ANOVA; *n* = 7, respectively; Figure 2D,E and Figure S1; Table 2). 67LR IgG also enhanced pERK1/2 level, but reduced AQP4 expression in the PC (*p* < 0.05 vs. control IgG, one-way ANOVA; *n* = 7, respectively; Figure 2D,E and Figure S1; Table 2).

SB202190 (a p38 MAPK inhibitor) co-treatment with 67LR IgG attenuated vasogenic edema induced by 67LR IgG infusion (*p* < 0.05 vs. vehicle, one-way ANOVA; *n* = 7, respectively; Figure 2A,B; Table 2), and inhibited p38 MAPK and PI3K/AKT phosphorylations (*p* < 0.05 vs. vehicle, one-way ANOVA; *n* = 7, respectively; Figure 2D,E and Figure S1; Table 2). However, SB202190 could not affect ERK1/2 phosphorylation and AQP4 expression in 67LR IgG-infused animals. These findings indicate that p38 MAPK activation may influence vascular permeability rather than AQP4 expression through the PI3K/AKT pathway following 67LR neutralization. 

Serum extravasation induced by rat 67LR IgG was also alleviated by co-treatment of wortmannin (a PI3K inhibitor), 3CAI (an AKT inhibitor) or U0126 (an ERK1/2 inhibitor) (*p* < 0.05 vs. vehicle, one-way ANOVA; *n* = 7, respectively; Figure 2A,B; Table 2). Co-administration of wortmannin inhibited the increased phosphorylations of PI3K/AKT and ERK1/2 induced by 67LR IgG infusion (*p* < 0.05 vs. vehicle, one-way ANOVA; *n* = 7, respectively; Figure 2D,E and Figure S1). Similarly, U0126 also decreased their phosphorylations (*p* < 0.05 vs. vehicle, one-way ANOVA; *n* = 7, respectively; Figure 2D,E and Figure S1; Table 2). 3CAI ameliorated only pAKT levels (*p* < 0.05 vs. vehicle, one-way ANOVA; *n* = 7, respectively; Figure 2D,E and Figure S1; Table 2). Although wortmannin, 3CAI, and U0126 did not affect p38 MAPK phosphorylation (Figure 2D,E), they increased AQP4 expression levels induced by 67LR neutralization (*p* < 0.05 vs. vehicle, one-way ANOVA; *n* = 7, respectively; Figure 2D,E, and Figure S1; Table 2). These findings indicate that PI3K and ERK1/2 may reciprocally regulate each phosphorylation and that AKT may be one of the common down-stream molecules for PI3K and ERK1/2 signaling pathways regulating serum extravasation and AQP4 expression following 67LR neutralization.

### 3.2. The Effects of 67LR Neutralization on p-p38 MAPK, pAKT and pERK1/2 Levels in Endothelial Cells and Astrocytes

**Figure 3:**
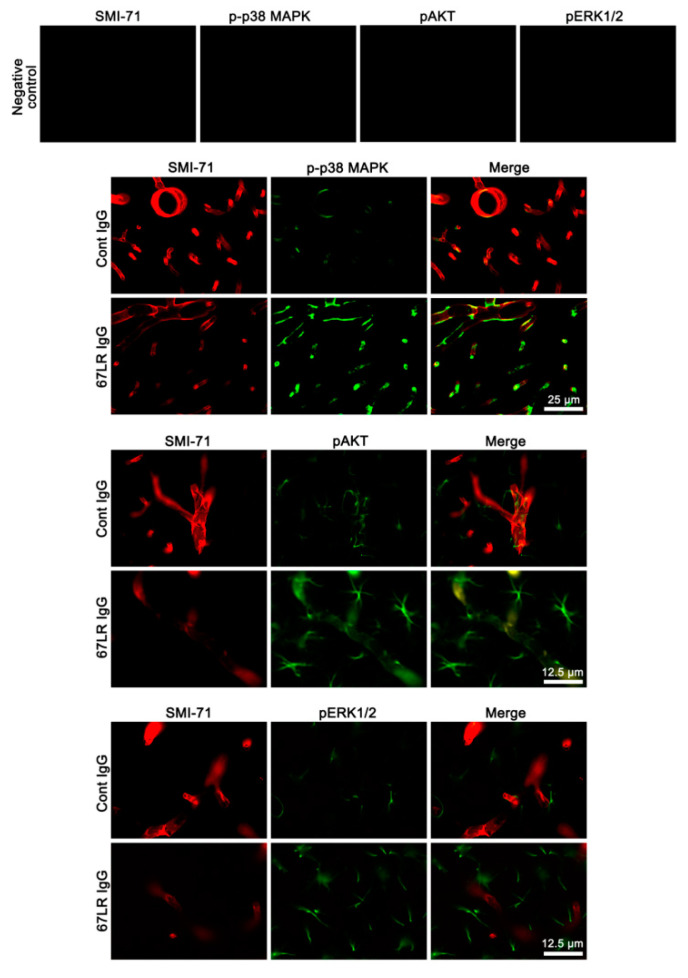
Localizations of SMI-71, p-p38 MAPK, pAKT, and pERK1/2 expression in the PC following 67LR neutralization. Up-regulations of p-p38 MAPK and pAKT expression are observed in endothelial cells following 67LR IgG infusion, accompanied by reduced SMI-71 expression. However, the pERK1/2 signal is rarely detected in endothelial cells.

**Figure 4:**
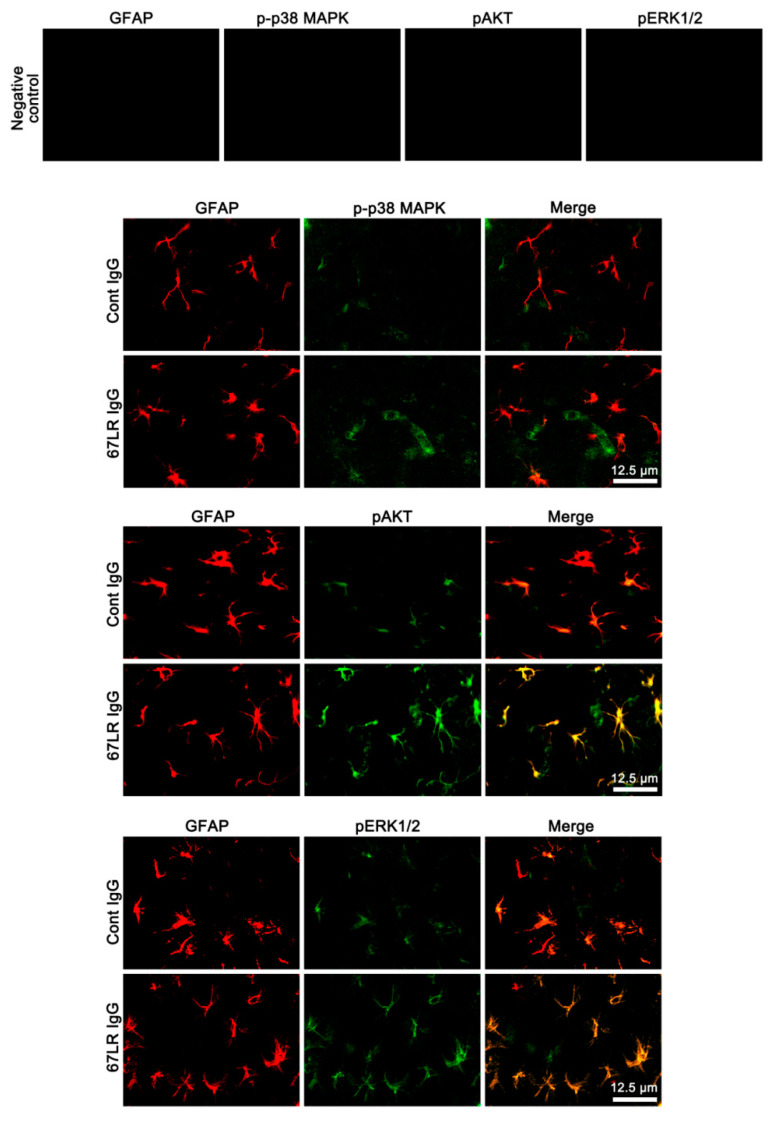
Localizations of p-p38 MAPK, pAKT, and pERK1/2 expression in the PC following 67LR neutralization. Up-regulations of pAKT and pERK1/2 expression are observed in astrocytes following 67LR IgG infusion. However, the p-p38 MAPK signal is rarely detected in astrocytes.

Since 67LR is identified in astrocytes and vascular endothelial cells [17,18,33,34], 67LR IgG infusion may likely affect p38 MAPK, AKT, and ERK1/2 phosphorylations in these cell populations. To identify the cellular profiles of the altered p38 MAPK, AKT, and ERK1/2 phosphorylations induced by 67LR neutralization, we performed the double immunofluorescent study. 67LR IgG infusion enhanced p-p38 MAPK and pAKT, but not pERK1/2, levels in endothelial cells, accompanied by the reduced SMI-71 (an endothelial BBB marker) expression (Figure 3). However, 67LR neutralization increased pAKT and pERK1/2, but not p-p38 MAPK, levels in astrocytes (Figure 4). Together with Western blot data (Figure 2D,E and Table 2), these findings indicate that 67LR IgG infusion may lead to p38 MAPK- and ERK1/2-mediated AKT activations in endothelial cells and astrocytes, respectively.

### 3.3. The Effects of SE on AQP4 Expression and Activities of p38 MAPK, PI3K/AKT, and ERK1/2

**Figure 5:**
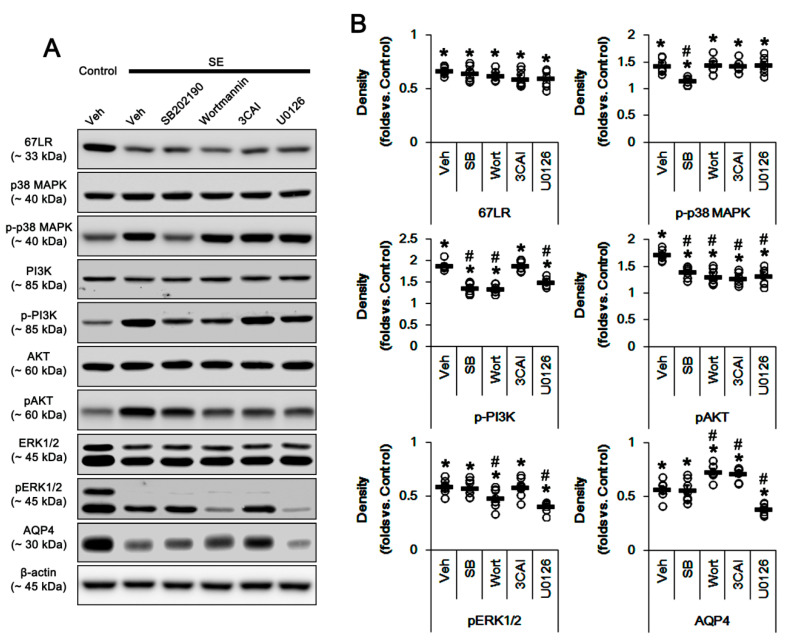
Effects of kinase inhibitors on protein phosphorylations and AQP4 expression in the PC following SE. SE decreased 67LR and AQP4 expressions and pERK1/2 level in the PC, while it increases p38 MAPK, PI3K, and AKT phosphorylations. SB202190, wortmannin, 3CAI, and U0126 do not affect 67LR expression following SE. SB202190 attenuates p38 MAPK and PI3K/AKT phosphorylations. Wortmannin, 3CAI, and U0126 do not affect p38 MAPK phosphorylation. Wortmannin and U0126 reduce PI3K/AKT and ERK1/2 phosphorylations. 3CAI inhibits only AKT phosphorylation. Wortmannin and 3CAI mitigate AQP4 expression, while U0126 deteriorates it. (**A**) Representative Western blot images for expressions and phosphorylations of 67LR, p38 MAPK, PI3K, AKT, ERK1/2, and AQP4. (**B**) Quantitative values (mean ± S.E.M) of the Western blot data concerning expressions and phosphorylations of 67LR, p38 MAPK, PI3K, AKT, ERK1/2, and AQP4 (*n* = 7, respectively). Open circles indicate each individual value. Horizontal bars indicate the mean value. Error bars indicate S.E.M. Significant differences are *^,#^ *p* < 0.05 vs. control (non-SE) animals and vehicle, respectively (one-way ANOVA).

Since SE reduces 67LR expression accompanied by p38 MAPK activation and the reduced AQP4 expression [17,18], we confirmed the effects of SE on PI3K, AKT, and ERK1/2 phosphorylations. SE decreased 67LR expression to 0.67-fold of control level in the PC (*p* < 0.05 vs. control animals, one-way ANOVA; *n* = 7, respectively; Figure 5A,B and Supplementary Figure S2; Table 3). However, SE increased phosphorylations of p38 MAPK, PI3K and AKT (*p* < 0.05 vs. control animals, one-way ANOVA; *n* = 7, respectively; Figure 5A,B and Supplementary Figure S2; Table 3), while it reduced pERK1/2 level and AQP4 expression (*p* < 0.05 vs. control animals, one-way ANOVA; *n* = 7, respectively; Figure 5A,B and Supplementary Figure S2; Table 3).

SB202190, wortmannin, 3CAI, and U0126 did not affect 67LR expression following SE (Figure 5A,B). SB202190 attenuated p38 MAPK and PI3K/AKT phosphorylations following SE (*p* < 0.05 vs. vehicle, one-way ANOVA; *n* = 7, respectively; Figure 5A,B and Supplementary Figure S2; Table 3). Wortmannin, 3CAI, and U0126 did not affect p38 MAPK phosphorylation following SE (Figure 5A,B; Table 3). Wortmannin and U0126 reduced PI3K/AKT and ERK1/2 phosphorylations following SE, respectively (*p* < 0.05 vs. vehicle, one-way ANOVA; *n* = 7, respectively; Figure 5A,B and Supplementary Figure S2; Table 3). 3CAI inhibited only SE-induced up-regulation of AKT phosphorylation (*p* < 0.05 vs. vehicle, one-way ANOVA; *n* = 7, respectively; Figure 5A,B and Supplementary Figure S2; Table 3). Wortmannin and 3CAI mitigated SE-induced reduction in AQP4 expression, while U0126 deteriorated it (*p* < 0.05 vs. vehicle, one-way ANOVA; *n* = 7, respectively; Figure 5A,B and Supplementary Figure S2; Table 3). Considering the data obtained from 67LR neutralization, these findings indicate that SE-induced down-regulation of 67LR expression may be relevant to p38 MAPK and PI3K/AKT activations, although total ERK1/2 activity was reduced. Furthermore, the increased AKT activity may reduce AQP4 expression following SE.

### 3.4. The Effects of SE on p-p38 MAPK, pAKT, and pERK1/2 Levels in Endothelial Cells and Astrocytes

**Figure 6:**
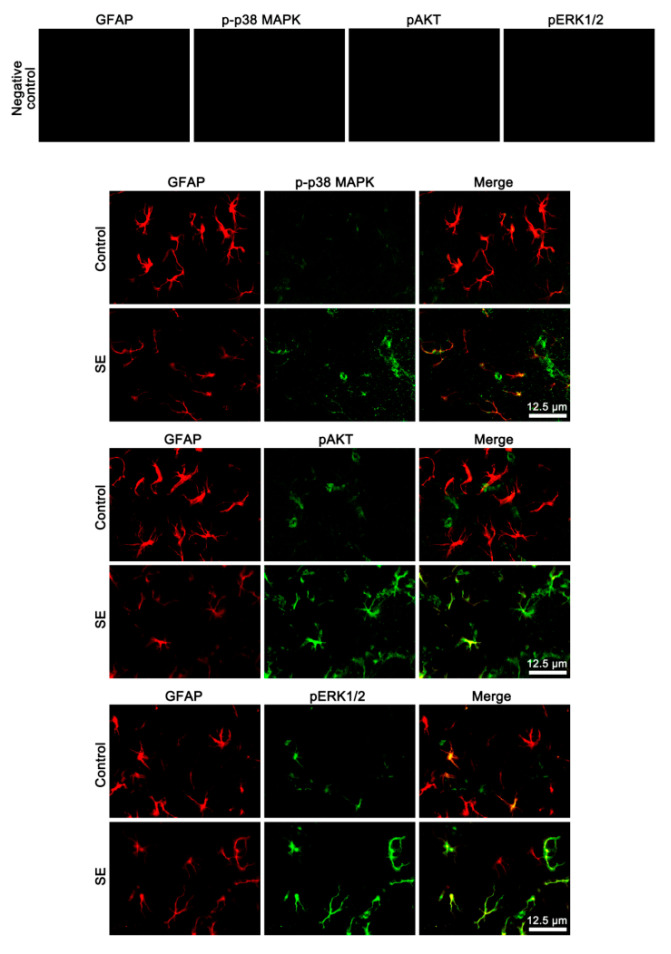
Localizations of p-p38 MAPK, pAKT, and pERK1/2 expression in the PC following SE. Upregulation of pAKT expression is observed in endothelial cells and astrocytes following SE. p-p38 MAPK and pERK1/2 signals are detected in endothelial cells and astrocytes, respectively.

**Figure 7:**
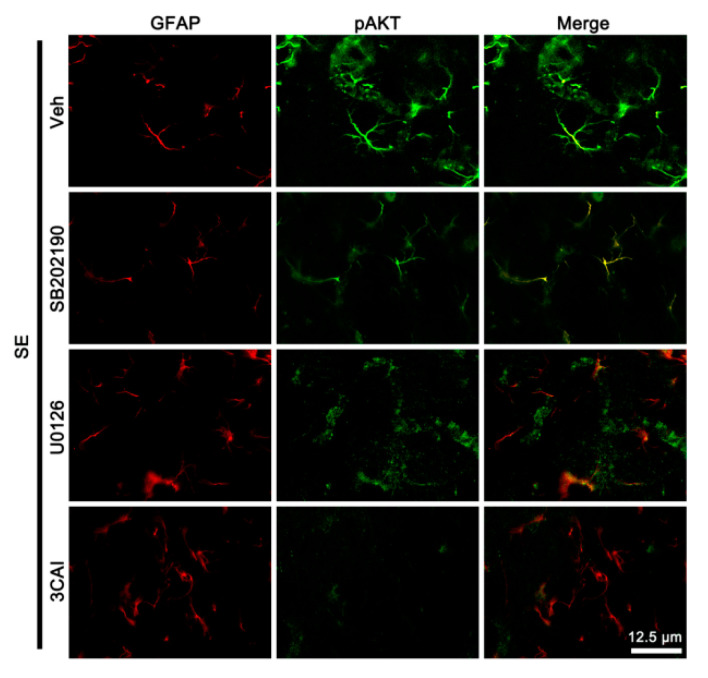
The effects of kinase inhibitors on localizations of pAKT expression in the PC following SE. Compared to the vehicle, SB202190 and U0126 reduce pAKT signal in endothelial cells and astrocytes, respectively. 3CAI diminishes it in both endothelial cells and astrocytes.

Next, we explored whether SE influences p38 MAPK, AKT, and ERK1/2 phosphorylations in cellular specific manners. Following SE, p-p38 MAPK signal was preferentially upregulated in endothelial cells (Figure 6). Furthermore, pERK1/2 signals were enhanced in remaining (surviving) astrocytes (Figure 6), although its total level was reduced on Western blot (Figure 5A,B). pAKT signal was increased in both astrocytes and endothelial cells (Figure 6). SB202190 and U0126 abrogated the up-regulated pAKT level in endothelial cells and astrocytes, respectively (Figure 7). 3CAI abolished it in both cells following SE (Figure 7). Thus, our findings suggest that SE-induced activations of 67LR-p38 MAPK-PI3K and 67LR-ERK1/2-PI3K signaling pathways may increase AKT activity in endothelial cells and astrocytes, respectively.

### 3.5. The Effects of p38 MAPK, AKT, and ERK1/2 Inhibitors on Serum Extravasation and Astroglial Loss Following SE

**Figure 8:**
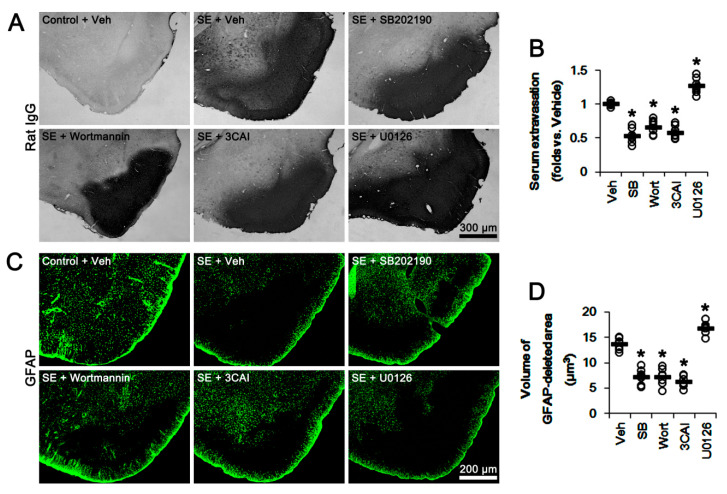
Effects of kinase inhibitors on vasogenic edema and astroglial degeneration in the PC following SE. SE evokes severe vasogenic edema and massive astroglial loss in the PC. As compared to vehicle, SB202190, wortmannin, and 3CAI attenuate serum extravasation and astroglial degeneration. However, U0126 deteriorates them. (**A**) Representative photographs for serum extravasation in the PC following SE. (**B**) Quantitative values (mean ± S.E.M) of serum extravasation following SE (*n* = 7, respectively). Open circles indicate each individual value. Horizontal bars indicate the mean value. Error bars indicate S.E.M. Significant differences are * *p* < 0.05 vs. vehicle (one-way ANOVA). (**C**) Representative photographs of astroglial loss in the PC induced by SE. (**D**) Quantitative values (mean ± S.E.M) of the volume of GFAP-deleted area following SE (*n* = 7, respectively). Open circles indicate each individual value. Horizontal bars indicate the mean value. Error bars indicate S.E.M. Significant differences are * *p* < 0.05 vs. vehicle (one-way ANOVA).

Finally, we evaluated the effects of kinase inhibitors on vasogenic edema formation and astroglial loss induced by SE. Unlike 67LR neutralization, SE resulted in astroglial loss in the PC, concomitant with severe vasogenic edema (*p* < 0.05 vs. control animals, one-way ANOVA; *n* = 7, respectively; Figure 8A–D). SB202190, wortmannin and 3CAI attenuated vasogenic edema and astroglial degeneration induced by SE (*p* < 0.05 vs. vehicle, one-way ANOVA; *n* = 7, respectively; Figure 8A–D). However, U0126 exacerbated serum extravasation and astroglial loss induced by SE (*p* < 0.05 vs. vehicle, one-way ANOVA; *n* = 7, respectively; Figure 8A–D). These findings indicate that 67LR-p38 MAPK-PI3K-AKT activation in endothelial cells may increase vascular permeability. In contrast, 67LR-ERK1/2-PI3K-AKT signaling pathways in astrocytes may regulate astroglial viability and AQP4 expression following SE.

## 4. Discussion

**Figure 9:**
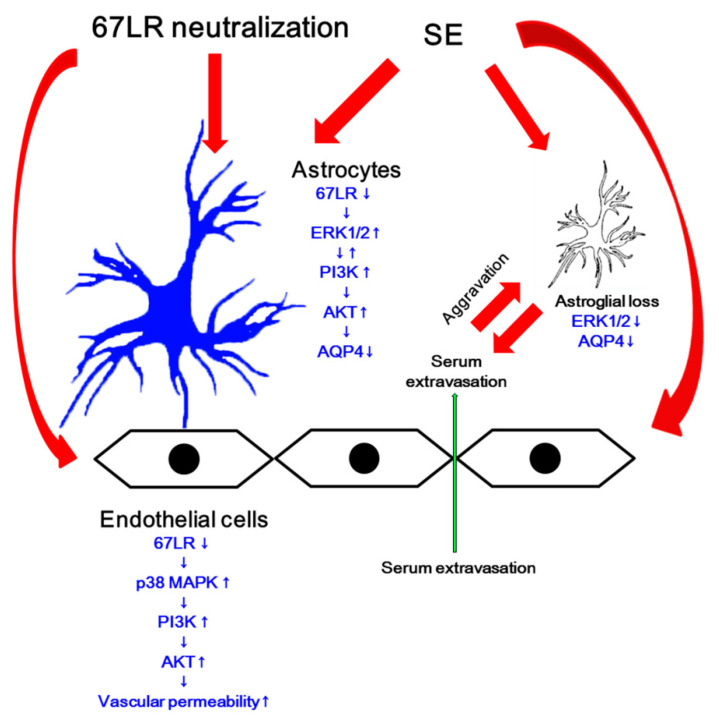
Scheme of role of 67-kDa LR in vasogenic edema formation and AQP4 expression. Blockade of 67LR induced by its neutralization and SE increases p38 MAPK and ERK1/2 phosphorylations in endothelial cells and astrocytes, respectively. Subsequently, both kinases activate PI3K/AKT signaling pathway. Increased AKT activity enhances endothelial permeability and triggers astroglial loss. However, AKT activation reduces AQP4 expression in astrocytes, which worsens vasogenic edema.

The major findings in the present study are that the blockade of 67LR functions leads to serum extravasation and the reduced AQP4 expression via two different PI3K/AKT-mediated pathways: p38 MAPK- and ERK1/2-mediated PI3K/AKT activation in endothelial cells and astrocytes, respectively (Figure 9). 

Vasogenic edema results from BBB breakdown, which increases capillary permeability and intracranial pressure. In addition, the leakage of blood-derived molecules during serum extravasation evokes neuronal hyperexcitability and neuroinflammatory responses [35,36,37,38]. Therefore, vasogenic edema is one of the risk factors inducing undesirable secondary complications in various neurological diseases. Recently, we have reported that blockade of 67LR functionality is involved in serum extravasation through p38 MAPK-VEGF signaling pathway. Furthermore, 67LR neutralization decreases AQP4 expression and enhanced ERK1/2 phosphorylation, which are reversed by U0126 [17,18]. As AQP4 deletion or its inhibition worsens vasogenic edema [10,11,12], we have speculated that 67LR neutralization-induced ERK1/2 activation would down-regulate AQP4 expression. Indeed, inhibition of 67LR expression increases basal ERK1/2 phosphorylation level, and soluble laminin decreases ERK1/2 phosphorylation [2]. In addition, epigallocatechin-3-gallate (EGCG, a green tea polyphenol), another ligand of 67LR [39], reduced ERK1/2 activity [40,41,42,43]. However, the discrepancies of ERK1/2-mediated AQP4 regulation have been reported: ERK1/2 activation decreases [23], increases [24], or cannot affect AQP4 expression [25]. With respect to these previous reports, it is plausible that ERK1/2 activation induced by 67-kDa LR neutralization may affect AQP4 expression mediated by unknown pathways. In the present study, we found that 67LR IgG infusion increased total ERK1/2 phosphorylation and astroglial pERK1/2 signal. SE also enhanced astroglial pERK1/2 level, in spite of the reduced total ERK1/2 phosphorylation. Furthermore, 67LR neutralization diminished AQP4 expression, which was abrogated by wortmannin, 3CAI and U0126. Interestingly, U0126 and wortmannin decreased total PI3K/AKT and ERK1/2 phosphorylations following SE and 67LR neutralization. The present data also demonstrate that U0126 and 3CAI reduced astroglial pAKT levels following SE. These findings indicate that AKT may be a common down-stream effector for PI3K- and ERK1/2-mediated AQP4 regulation. Indeed, AKT activation inhibits AQP4 expression by increasing Foxo3a activity [16]. Considering the interaction between PI3K and ERK1/2 pathways [26,27,28], the differential reciprocal modulation of PI3K and ERK1/2 in response to stimuli or the presence of this crosstalk may result in the discrepancies of ERK1/2-mediated AQP4 regulation. Regardless of interaction between PI3K and ERK1/2, our findings suggest that AKT activation may down-regulate AQP4 expression in astrocytes following SE and 67LR neutralization.

Givant-Horwitz et al. [32] reported that 67LR is involved in laminin-mediated p38 MAPK activation, which participates in SE-induced vasogenic edema via the increased PI3K/AKT-mediated eNOS expression [14,16]. Furthermore, 67LR neutralization activates p38 MAPK-mediated VEGF expression, which also increases vascular permeability [17]. Indeed, SB202190 alleviates serum extravasation induced by SE and 67LR IgG infusion, while it does not affect AQP4 expression [17,18]. Consistent with these reports, the present study reveals that 67LR neutralization and SE increased p38 MAPK-mediated PI3K/AKT phosphorylations. Unlike the case of ERK1/2 phosphorylation, however, the up-regulated p-p38 MAPK level was restricted to endothelial cells showing the decreased SMI-71 expression. Furthermore, SB202190 and 3CAI mitigated serum extravasation and astroglial loss, accompanied by the decreased pAKT level in endothelial cells, but not astrocytes, following SE. Since SE-induced vasogenic edema results in extensive astroglial loss that subsequently aggravates serum extravasation due to the impairment of AQP4-dependent water elimination [12], these findings indicate that 67LR-p38 MAPK-PI3K/AKT signaling pathway may increase vascular permeability due to BBB disruption, which subsequently evokes astroglial loss. Therefore, our findings suggest that AKT may be one of the important molecules regulating astroglio-endothelial interactions during vasogenic edema formation. 

In the present study, 67LR neutralization increased total ERK1/2 phosphorylation. Furthermore, U0126 abrogated serum extravasation and the down-regulation of AQP4 expression induced by 67LR neutralization. In contrast, SE decreased total ERK1/2 phosphorylation, and U0126 deteriorated vasogenic edema and AQP4 down-regulation following SE. Thus, it would contradict the role of 67LR dysfunction in ERK1/2-mediated AQP4 regulation. Unlike 67LR IgG infusion, SE leads to acute and devastating astroglial degenerations that are characterized by a pattern of selective vulnerability [12,27,44,45,46,47]. Thus, it is likely that the SE-induced astroglial damage may decrease the total ERK1/2 phosphorylation and AQP4 expression. However, immunohistochemical study revealed that pERK1/2 signals were enhanced in remaining (surviving) astrocytes following SE, although total ERK1/2 phosphorylation was reduced on Western blot. Therefore, our findings indicate that SE-induced 67LR dysfunction may increase ERK1/2 activity in remaining astrocytes, which would reduce AQP4 expression. As ERK1/2 itself modulates the cell cycle re-entry and cell survival [48,49,50], ERK1/2 inhibition abrogates astroglial proliferation and up-regulation of GFAP expression, which are hallmarks of reactive astrogliosis [51,52]. Indeed, U0126 decreases astroglial viability [53,54,55], and aggravates vasogenic edema following SE [12,13]. Considering the role of AQP4 in vasogenic edema formation [10,11,12] and absence of astroglial loss induced by 67LR IgG infusion in the present study, it is likely that U0126 may exacerbate SE-induced serum extravasation and the down-regulated AQP4 expression by decreasing astroglial viability, unlike 67LR neutralization.

On the other hand, some AQP4 isoforms are reported: AQP4a (M1), AQP4b, AQP4c (M23), AQP4d, AQP4e, and AQP4f in the brain [56,57,58,59]. Among them, AQP4a, AQP4c, and AQP4e are water-permeable and sensitive to changes in extracellular osmolality [60,61,62], which alternatively splice into AQP4b, AQP4d, and AQP4f isoforms, respectively [63]. Furthermore, De Bellis et al. [64] have reported a newly characterized AQP4ex isoform that is generated by translational readthrough. AQP4ex contains a 29 amino acid *C*-terminal extension, which is involved in proper membrane localization of AQP4 and interaction with other intracellular proteins. As AQP4ex display a perivascular polarization and expression in dystrophin-dependent pools, it is necessary for anchoring of the perivascular AQP4. Indeed, the absence of AQP4ex isoform, AQP4 assemblies are mislocalized in the brain [64,65]. In the present study, we could not explore the alterations in AQP4 isoforms induced by 67LR neutralization and SE, since the commercial antibodies for AQP4 isoforms are unavailable. However, Cartagena et al. [66] have reported that in the penetrating ballistic-like brain injury (PBBI) model AQP4a (M1) decreases at 3 and 7days post-injury, while AQP4c (M23) levels were highly variable with no significant changes. AQP4ex also regulates water transport at the BBB level, and binds with human neuromyelitis optica autoantibody that is associated with autoimmune demyelinating diseases in the central nervous system [67]. In addition, the deletion of gap junction forming proteins connexin-43 (Cx43) and connexin-30 (Cx30) increases AQP4a (M1) and AQP4ex isoform levels, but reduces AQP4c (M23) isoform in astrocytes [68]. Considering these reports, further studies are needed to validate AQP4 isoforms affected by 67LR functions in astroglio-vascular interactions. 

## 5. Conclusions

To the best of our knowledge, the present data reveal for the first time that 67LR affected the severity of vasogenic edema formation in two different signaling pathways. Blockade of 67LR functions activated p38 MAPK-PI3K/AKT axis, which increased vascular permeability. It also increased ERK1/2- and PI3K-mediated AKT phosphorylation in astrocytes, which decreased AQP4 expression (Figure 9). Thus, our findings suggest that the regulation of 67-kDa LR expression/functions may be one of the considerable factors for medication of vasogenic edema formation and prevention of its complications.

## Figures and Tables

**Table 1:** Primary antibodies and lectin used in the present study.

Antigen	Host	Manufacturer (Catalog Number)	Dilution Used
AKT (a synthetic peptide corresponding to the *C*-terminal sequence of mouse AKT)	Rabbit IgG	Cell signaling (#9272)	1:1000 (WB)
GFAP (Purified glial filament)	Mouse IgG	Millipore (#MAB3402)	1:5000 (IH)
p38 MAPK (a synthetic peptide corresponding to the sequence of human p38 MAPK)	Rabbit IgG	Cell signaling (#9212)	1:1000 (WB)
p-p38 MAPK (a KLH conjugated synthetic phosphopeptide derived from human p38 MAPK around the phosphorylation site of T180/Y182)	Rabbit IgG	Abbiotec (#251246)	1:200 (WB) 1:50 (IH)
ERK1/2 (a synthesized peptide derived from human ERK 1/2 around the non-phosphorylation site of Y204)	Rabbit IgG	Biorbyt (Orb160960)	1:2000 (WB)
pERK1/2 (a phosphopeptide encompassing the TxY motif in the activation loop of ERK1/2)	Rabbit IgG	Millipore (#05-797R)	1:1000 (WB) 1:100 (IH)
AQP4 (a GST fusion protein corresponding to amino acid residues 249–323 of rat AQP4)	Rabbit IgG	Alomone labs (#AQP-004)	1:5000 (WB)
pAKT-T308 (a synthetic phosphopeptide corresponding to residues around Thr308 of mouse AKT)	Rabbit IgG	Cell signaling (#9275)	1:1000 (WB) 1:50 (IH)
pPI3K-Y458 (a synthetic phosphopeptide corresponding to residues surrounding Tyr458 of mouse p85)	Rabbit IgG	Cell signaling (#4228)	1:1000 (WB)
PI3K (a synthetic peptide corresponding to residues of human PI3 Kinase p85)	Rabbit IgG	Cell signaling (#4292)	1:1000 (WB)
Rat IgG	Goat IgG	Vector (#BA-9400)	1:200 (IH)
SMI-71 (rat BBB)	Mouse IgM	Covance (#SMI-71R)	1:1000 (IH)
67LR (a synthetic peptide corresponding to amino acid residues 250–350 of Human 67LR)	Rabbit IgG	Abcam (#ab133645)	1:1000 (WB)
β-actin (a KLH conjugated peptide of β-cytoplasmic actin *N*-terminal peptide)	Mouse IgG	Sigma (#A5316)	1:5000 (WB)

IH: Immunohistochemistry; WB: Western blot.

**Table 2:** Densities (mean ± S.D. fold of control IgG-infused animal level) of 67LR, total kinases, phospho (p)-kinases, and AQP4 in the PC of 67LR IgG-infused animals (*^,#^ *p* < 0.05 vs. control IgG and vehicle, respectively).

Inhibitor Proteins	Vehicle	SB202190	Wortmannin	3CAI	U0126
67LR	1.03 ± 0.03	1.04 ± 0.07	1.06 ± 0.05	1.05 ± 0.06	1.05 ± 0.09
P38 MAPK	1.03 ± 0.09	1.05 ± 0.08	0.97 ± 0.09	1.08 ± 0.07	1.06 ± 0.05
p-p38 MAPK	1.56 ± 0.15 *	1.16 ± 0.07 *^,#^	1.49 ± 0.11 *	1.48 ± 0.09 *	1.47 ± 0.13 *
PI3K	0.95 ± 0.08	0.94 ± 0.03	0.91 ± 0.09	0.98 ± 0.07	0.92 ± 0.11
p-PI3K	1.98 ± 0.19 *	1.20 ± 0.04 ^#^	1.25 ± 0.10 ^#^	2.06 ± 0.21 *	1.52 ± 0.11 *^,#^
AKT	0.99 ± 0.09	0.97 ± 0.08	1.00 ± 0.05	0.90 ± 0.11	0.92 ± 0.08
p-AKT	1.64 ± 0.10 *	1.26 ± 0.06 *^,#^	1.26 ± 0.15 *^,#^	1.23 ± 0.10 *^,#^	1.28 ± 0.14 *^,#^
ERK1/2	1.02 ± 0.06	1.04 ± 0.07	1.01 ± 0.04	1.10 ± 0.09	1.06 ± 0.07
p-ERK1/2	1.51 ± 0.08 *	1.44 ± 0.07 *	1.26 ± 0.09 *^,#^	1.44 ± 0.10 *	1.11 ± 0.05 *^,#^
AQP4	0.57 ± 0.10 *	0.51 ± 0.11 *	0.82 ± 0.08 *^,#^	0.81 ± 0.08 *^,#^	0.79 ± 0.10 *^,#^

**Table 3:** Densities (mean ± S.D. fold of control animal level) of 67LR, total kinases, phospho (p)-kinases, and AQP4 in the PC of SE-induced animals (*^,#^ *p* < 0.05 vs. control animal and vehicle, respectively).

Inhibitor Proteins	Vehicle	SB202190	Wortmannin	3CAI	U0126
67LR	0.67 ± 0.04 *	0.65 ± 0.07 *	0.63 ± 0.05 *	0.59 ± 0.08 *	0.61 ± 0.09*
P38 MAPK	1.08 ± 0.09	1.07 ± 0.09	1.09 ± 0.08	1.00 ± 0.07	1.01 ± 0.05
p-p38 MAPK	1.44 ± 0.13 *	1.16 ± 0.07 *^,#^	1.45 ± 0.14 *	1.43 ± 0.11 *	1.45 ± 0.15 *
PI3K	0.99 ± 0.04	1.01 ± 0.03	1.04 ± 0.07	0.96 ± 0.07	1.01 ± 0.08
p-PI3K	1.89 ± 0.10 *	1.38 ± 0.13 *^,#^	1.35 ± 0.10 *^,#^	1.89 ± 0.12 *	1.51 ± 0.10 *^,#^
AKT	1.08 ± 0.09	1.01 ± 0.08	1.05 ± 0.05	0.97 ± 0.11	1.05 ± 0.08
p-AKT	1.73 ± 0.10 *	1.40 ± 0.11 *^,#^	1.32 ± 0.15 *^,#^	1.28 ± 0.11 *^,#^	1.33 ± 0.16 *^,#^
ERK1/2	0.99 ± 0.06	1.06 ± 0.07	1.05 ± 0.04	0.98 ± 0.02	0.99 ± 0.03
p-ERK1/2	0.59 ± 0.06 *	0.58 ± 0.08 *	0.49 ± 0.09 *^,#^	0.59 ± 0.09 *	0.41 ± 0.05 *^,#^
AQP4	0.56 ± 0.08 *	0.56 ± 0.11 *	0.73 ± 0.07 *^,#^	0.71 ± 0.06 *^,#^	0.38 ± 0.05 *^,#^

## Supplementary Materials

The following are available online at https://www.mdpi.com/2073-4409/9/7/1670/s1, Figure S1: Representative full-gel images of Western blots in Figure 2, Figure S2: Representative full-gel images of Western blots in Figure 5.
