# Supplementary material for: Blockade of 67-kDa Laminin Receptor Facilitates AQP4 Down-Regulation and BBB Disruption via ERK1/2-and p38 MAPK-Mediated PI3K/AKT Activations
Source: Cells. 2020 Jul 11;9(7):1670. doi: 10.3390/cells9071670 (PMC7407797; doi:10.3390/cells9071670)
Supplement: Supplementary file 1 [file cells-09-01670-s001.pdf]

Supplementary information

Blockade of 67-kDa laminin receptor facilitates AQP4 down-regulation and BBB disruption via ERK1/2- and p38 MAPK-mediated PI3K/AKT activations

Ji-Eun Kim[1,2], Hana Park[1,2], Ji-Eun Lee[1,2], Tae-Cheon Kang[1,2*]

[1]Department of Anatomy and Neurobiology, College of Medicine, Hallym University, Chuncheon 24252, South Korea

[2]Institute of Epilepsy Research, College of Medicine, Hallym University, Chuncheon 24252, South Korea

Running title: 67-kDa LR-mediated regulation of BBB disruption and AQP4 down-regulation

* Correspondence to: T. -C. Kang, Department of Anatomy and Neurobiology, College of Medicine, Hallym University, Chuncheon, Kangwon-Do 24252, South Korea; Tel: +82-33-248-2524; Fax: +82-33-248-2525; E-mail: tckang@hallym.ac.kr

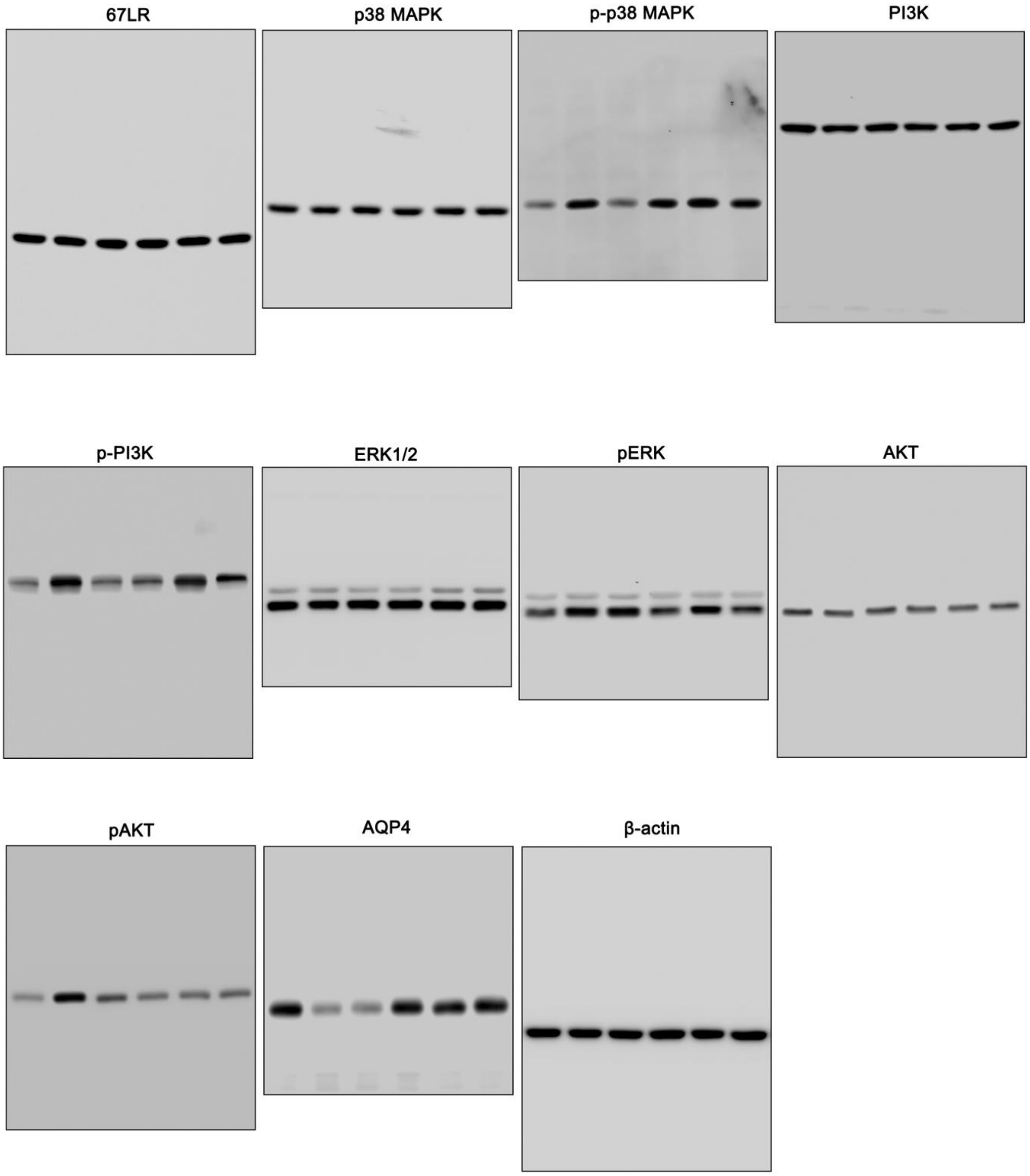

Supplementary Figure 1. Representative full-gel images of Western blots in Figure 2.

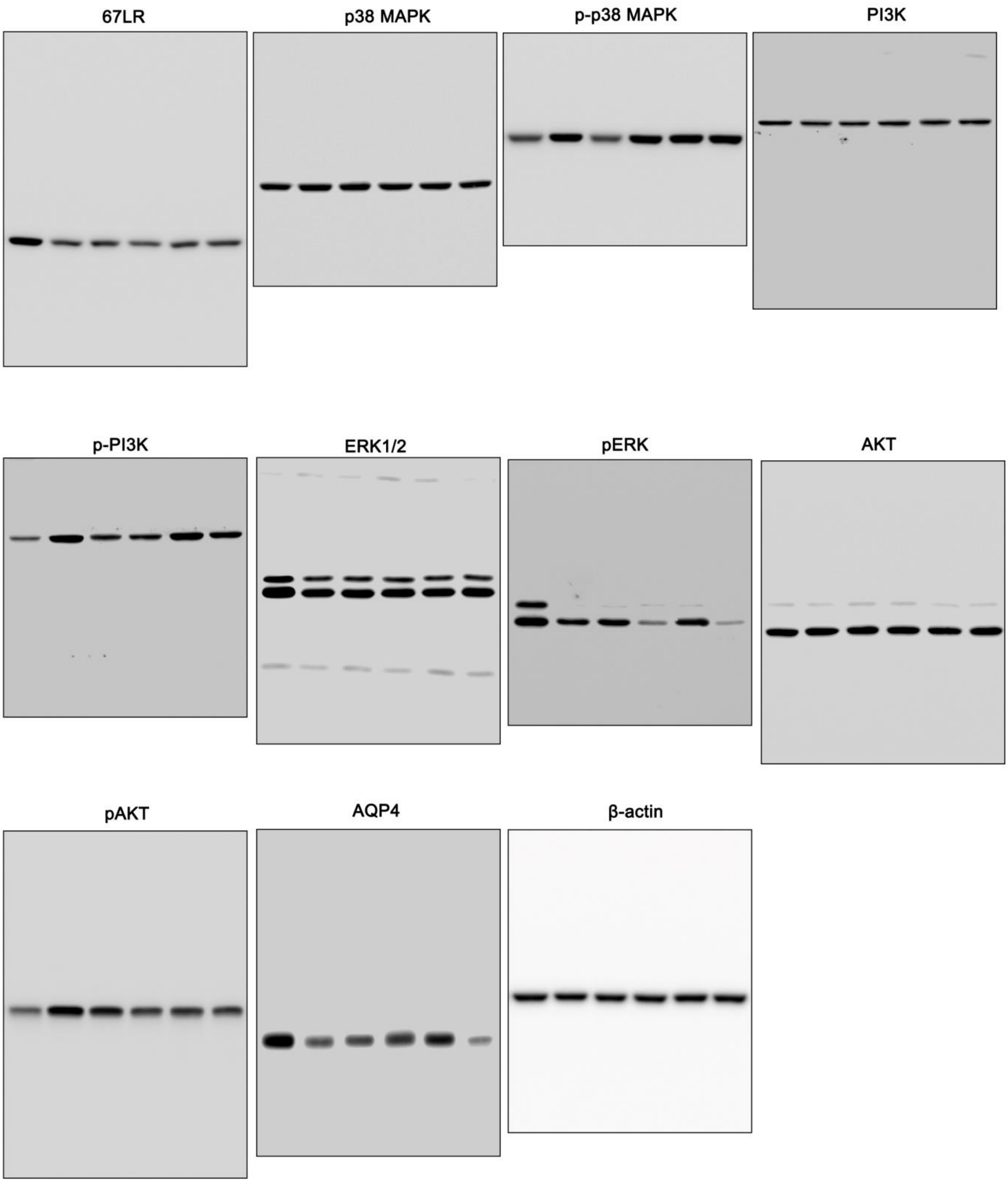

Supplementary Figure 2. Representative full-gel images of Western blots in Figure 5.
